# Prenatal exposure to selenium may protect against wheezing in children by the age of 3

**DOI:** 10.1002/iid3.138

**Published:** 2016-12-11

**Authors:** Nour Baïz, Julie Chastang, Gladys Ibanez, Isabella Annesi‐Maesano

**Affiliations:** ^1^ Epidemiology of Allergic and Respiratory Diseases Department (EPAR)Sorbonne UniversitésUPMC Univ Paris 06INSERM, Institut Pierre Louis d'Epidémiologie et de Santé Publique (IPLESP UMRS 1136)Medical School Saint‐Antoine75012 ParisFrance; ^2^ Department of General PracticeUPMC Université Paris 6Medical School Saint Antoine75012 ParisFrance

**Keywords:** Asthma, allergic diseases, gestation, mother–child cohort, selenium, wheezing

## Abstract

**Introduction:**

It has been suggested that human in utero exposure to heavy metals such as selenium can reduce the prevalence of childhood asthma and allergic diseases. However, data on this topic are scarce. The objective of the present study was to assess the putative associations between maternal selenium level during pregnancy and the risk of asthma, wheezing, allergic rhinitis, and atopic dermatitis in children from the EDEN birth cohort by the age of 1 and 3 years.

**Methods:**

Plasma selenium concentrations were measured in maternal blood during mid‐pregnancy (24–28 weeks of gestation) in 861 mothers. Cohort children were followed up from birth to 3 years using health questionnaires filled out by the parents for asthma, wheezing, allergic rhinitis, and atopic dermatitis. Maternal plasma selenium was related to the childhood outcomes by the age of 1 and 3 years.

**Results:**

Our results showed a significant negative association between a high maternal plasma selenium level during pregnancy and the risk of wheezing in the child by the age of 1 and 3 years. However, maternal plasma selenium during pregnancy was not associated with the prevalence of asthma, allergic rhinitis or atopic dermatitis.

**Conclusions:**

The results of this study suggest that the level of fetal exposure to maternal selenium could have an influence on the risk of wheezing in infancy and potentially on the risk of developing asthma later in life.

## Introduction

Over the last 20 years, the prevalence of asthma and allergic disease has increased in most developed countries 1, 2. It is becoming increasingly clear that exposure to certain factors in utero and/or during the first few years after birth can influence the development of asthma and allergic diseases, and thus might explain the observed increase in prevalence.

We are all exposed to many chemicals present in the environment, which enter the body by inhalation, food consumption and/or skin contact. Some studies have found that maternal exposure to heavy metals during pregnancy influences the newborn's height, weight 3, neurological development 4, and immune development 5. Other studies have demonstrated that the fetus is indeed exposed to the environmental and dietary pollutants with which the mother is in contact during pregnancy. Indeed, it has been proven that heavy metals, including selenium, cross the placental barrier 6, 7.

It has been suggested that heavy metals such as selenium 8, 9, 10 which are mainly ingested in food) reduce the risk of developing asthma and allergic diseases. However, data on this topic are scarce. When designing the present study, we hypothesized that high fetal exposure to selenium could decrease the risk of asthma and common allergic diseases in the child.

Hence, the objective of the present study was to examine the association between maternal level of plasma selenium during pregnancy and the risk of asthma, wheezing, allergic rhinitis, and atopic dermatitis in children from the EDEN birth cohort by the age of 1 and 3 years of age.

## Materials and Methods

### The study population

Mother–child pairs were recruited in the EDEN (Etude des Déterminants pré et post natals du développement et de la santé de l′Enfant) Prospective Birth Cohort Study 11. Two thousand and two pregnant women were recruited from February 2003 to January 2006, before their 24th week of gestation in two French university hospitals in the cities of Poitiers and Nancy 11. In total, 1899 mother–child pairs with complete data at birth were available. Our investigation was conducted in 861 mother‐newborn pairs for whom a maternal blood sample collected during pregnancy was available, for whom selenium in plasma was measured and who completed the health questionnaires up to age 3.

### Ethics statement

The study received approval from the ethics committee (CCPPRB) of Kremlin Bicêtre on 12 December 2002 and from CNIL (Commission Nationale Informatique et Liberté), the French data privacy institution. The study was approved on 12 December 2002. Written consent was obtained from the mother for herself at the beginning of the study and from both parents for the newborn child after delivery.

### Assays of maternal selenium concentration

Maternal samples of venous blood were collected between the 24th and 28th weeks of amenorrhea. EDTA tubes were used to collect blood for the plasma selenium assay. The blood tubes were prepared within an hour of sample collection. After aliquoting, all biological material was frozen at −80°C. After centrifugation at 3000 rpm for 5 min, maternal plasma was aliquoted and frozen at −80°C for later analysis. Maternal plasma selenium concentration (expressed in µg/L) was assayed using spectrofluorimetry with 2,3‐diaminonaphtalene; the latter compound binds selenium to form a fluorescent complex (excitation: 364 nm; emission: 523 nm). Plasma selenium data were available for 861 women.

### Study variables

#### Health‐related variables

The parents completed a questionnaire including questions on asthma and wheezing at the age of 1, 2 and 3, on allergic rhinitis at the age of 2 and 3, based on the validated phase I questionnaire from the International Study of Asthma and Allergies in Childhood (ISAAC) 1, and on doctor diagnosed atopic dermatitis. Asthma was defined as parental report of doctor‐diagnosis of asthma plus either one or more attacks of wheeze or asthma medication in the last 12 months. Wheeze was defined as present if the parents answered “yes” to the question “Has your child had wheezing or whistling in the chest in the preceding 12 months?”. Allergic rhinitis was defined as sneezing, nasal congestion, or rhinitis, other than with respiratory infections, accompanied by eye itching and tearing during the previous 12 months 12. Finally, parents reported atopic dermatitis diagnosed by a doctor. The responses at age of 1, 2, and 3 years were incorporated into calculated lifetime prevalence of wheezing, asthma, atopic dermatitis and allergic rhinitis at age 3.

#### Other variables

We collected information on potential confounding factors related to the health‐related variables in children, including the child's gender, birthweight, gestational age, season of birth, the number of older siblings (0, 1–2, ≥3), exclusive breastfeeding for at least 4 months, the mother's age when the child was born (classified as <25, 25–34, and >34 years), pre‐pregnancy maternal body mass index (BMI, classified as 18.5–24.9, 25.0–29.9, 30.0–34.9, and 35.0–39.9 kg/m^2^), parental history of allergy (physician‐diagnosed allergic diseases, including asthma, allergic rhinitis, atopic dermatitis and food allergies), the mother's and father's educational level (primary or below, secondary or tertiary, the household income (≤2300 euros per month vs. >2300 euros per month, based on the median income of the study population), the city of residence (Nancy or Poitiers), tobacco use during pregnancy, the child's exposure to tobacco smoke in the environment from 0 to 3 years of age and residence in damp housing from 0 to 3 years of age.

### Statistical analyses

Categorical variables were expressed as the number (%) and continuous variables were expressed as the mean ± standard deviation (SD). The study population was compared with the rest of the cohort without selenium measurements by applying a chi‐squared test (for categorical variables) or a Mann–Whitney U test (for continuous variables).

The mean selenium concentrations were compared according to maternal educational level, BMI, age, socio‐professional category and tobacco use during pregnancy by applying Student's test (for binary variables) or an analysis of variance (ANOVA, for factors with more than two modes).

We used logistic regression models to investigate the associations between health‐related variables and selenium exposure defined in tertiles (first tertile: low exposure, i.e., the reference value; 2nd tertile: moderate exposure; 3rd tertile: high exposure) or as a continuous variable. We estimated the odds ratio (OR) [95% confidence interval (CI)] for each health‐related variable in the child by the age of 1 and 3.

In order to identify potential confounding factors, bivariate analyses were performed for each health‐related variable. Firstly, we selected variables associated with a health event that had a *p*‐value <0.30. Secondly, factors that changed the value of the OR by at least 20% were selected and included in multivariate models. In addition to the confounding factors included in the models by virtue of their statistically significant association with a health‐related variable, we selected a number of adjustment variables on the basis of their documented relationship with asthma and allergic diseases (i.e., independently of their association with fetal exposure to heavy metals or with health‐related variables). These included the child's gender 13, 14, the pre‐pregnancy maternal BMI 15, the birthweight 16, the season of birth 17, 18, the number of older siblings 19, and exclusive breastfeeding for at least 4 months 20.

Hence, we adjusted the logistic regression models with the following factors: maternal age, pre‐pregnancy maternal BMI, maternal active smoking during pregnancy, environmental exposure of the child to tobacco smoke (from 0 to 3 years of age), residence in damp housing (from 0 to 3 years of age), maternal atopy, the child's gender, birthweight, season of birth, exclusive breastfeeding for at least 4 months, the number of older siblings, the mother's educational level and the household income. Adjustment for the father's atopic status or educational level did not modify the results significantly.

Lastly, to study the modulation of the effect of maternal selenium level on the child's health‐related variables by maternal atopy, we stratified the population by maternal history of asthma and allergies and tested the interaction terms (maternal selenium × maternal atopy). No effect modification by maternal atopy was observed (interaction terms were not significant).

All statistical analyses were performed using SAS software (version 9.3, SAS Institute Inc., Cary, NC). The threshold for statistical significance was set to *p* < 0.05.

## Results

### Characteristics of the study population

Table 1 presents the characteristics of the women and children in the main analysis (for whom selenium measurements were performed, *n* = 861) and those of who were not included (without selenium measurements, *n* = 1038). Our population did not differ significantly from the rest of the cohort with respect to all characteristics, except for the city of residence, and season of birth (Table 1).

**Table 1:** Characteristics of the mothers and newborns in our study population with selenium measurements (*n* = 861) and in the rest of the population without selenium measurement (*n* = 1038)

	Mean ± SD or frequency (%)		
Variable	Without data (*n* = 1038)	With data (*n* = 861)	*p*‐value^†^
Mothers			
Study centre, %			
Poitiers	53.49	45.79	
Nancy	46.51	54.21	**0.0008**
Age (years), mean ± SD	30.22 ± 4.82	29.72 ± 4.96	0.07
<25, %	13.44	17.06	
25–34, %	69.48	68.35	
>34, %	17.08	14.59	0.10
BMI (kg/m^2^), mean ± SD	26.40 ± 4.58	26.29 ± 4.47	0.72
Normal, %	45.20	45.76	
Overweight, %	37.54	37.18	
Moderately obese, %	11.83	12.00	0.96
Severely obese, %	5.43	5.06	
Educational level, %			
Primary or below	5.93	7.96	
Secondary	60.80	62.35	
Tertiary	33.27	29.69	0.10
Household income >2300 euros per month, %	55.83	49.94	0.06
Tobacco use during pregnancy, %	27.00	31.05	0.11
Maternal atopy	31.93	32.71	0.72
Duration of pregnancy (WA), mean ± SD	39.27 ± 1.69	39.17 ± 1.80	0.24
Newborns			
Gender (female), %	48.09	46.66	0.54
Birthweight (kg), mean ± SD	3.28 ± 0.50	3.28 ± 0.52	0.71
Premature birth, %^‡^	5.64	6.32	0.54
Presence of siblings, %			
0	46.65	46.16	
1–2	48.28	49.65	
≥3	5.07	4.19	0.61
Season of birth, %			
Summer	24.79	32.75	
Autumn	21.89	20.82	
Winter	23.23	19.06	
Spring	30.11	27.37	**0.001**
Exclusive breastfeeding for at least 4 months, %	13.39	13.39	0.82

*WA, weeks of amenorrhea.

^†^The *p*‐value in a chi‐squared test (for categorical variables) or a Mann–Whitney U test (for continuous variables), statistically significant p‐values are displayed in bold.

^‡^<37 weeks of amenorrhea.

**Figure 1:**
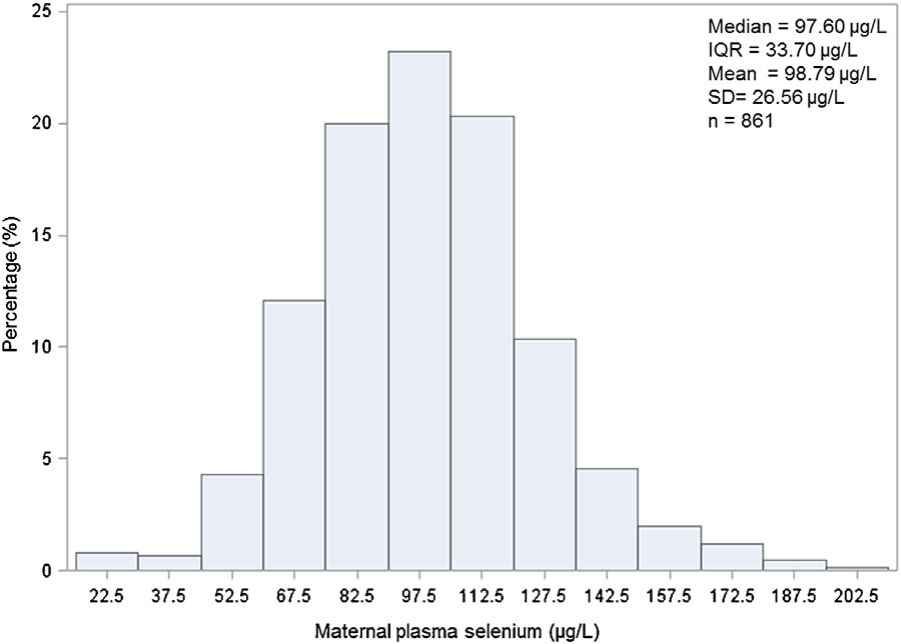
Distribution of selenium levels in maternal plasma (μg/L). Plasma selenium levels are indicated on the x‐axis and percentages of mothers are indicated on the y‐axis. IQR, interquartile range; SD, standard deviation.

Figure 1 shows the distribution of maternal plasma selenium concentration. The median level and interquartile range (IQR) were 97.6 and 33.70 µg/L. The mean plasma selenium concentration and standard deviation were 98.79 and 26.56 µg/L.

By the age of 1, 6.73% of the children had asthma, 22.69% had wheezing, and 26.48% had atopic dermatitis. By the age of 3, 9.23% of the children had current or previous asthma, 33.98% had wheezing, 10.86% had allergic rhinitis, and 32.40% had atopic dermatitis.

### Relationships between maternal characteristics and selenium level

The mean plasma selenium concentration was 97.50 µg/L in Poitiers and 100.25 µg/L in Nancy (Table 2). Women aged more than 34 years had a slightly higher mean concentration of selenium than in younger women, and women who never smoked during pregnancy presented a slightly higher mean concentration than those who did, although the differences were not statistically significant.

**Table 2:** Selenium concentration and selected maternal characteristics

	Maternal plasma selenium concentration (μg/L)	
Variable	Mean ± SD*	p**
Study centre		
Poitiers	97.50 ± 24.95	
Nancy	100.25 ± 28.29	0.05
Educational level		
Primary or below	94.4 ± 23.91	
Secondary	98.73 ± 26.95	
Tertiary	100.67 ± 26.96	0.14
BMI		
Normal	100.24 ± 26.59	
Overweight	97.91 ± 27.30	
Moderately obese	95.84 ± 25.84	
Severely obese	101.38 ± 23.79	0.38
Age (years)		
<25	94.46 ± 25.91	
25–34	99.67 ± 26.45	
>34	101.68 ± 26.52	0.06
Maternal atopy		
No	99.77 ± 27.65	
Yes	96.41 ± 23.77	0.32
Tobacco use during pregnancy		
No	99.67 ± 26.24	
Yes	96.81 ± 27.25	0.06
Household income		
≤2300 euros per month	98.20 ± 25.47	
>2300 euros per month	99.38 ± 27.63	0.28

*SD, standard deviation.

**The *p*‐value in a Student's test or an ANOVA.

### Associations between maternal selenium level and health‐related variables in the child by the age of 1 and 3

An analysis of the associations between selenium levels (in tertiles) and the health‐related variables revealed a significant, negative association between a high maternal plasma selenium level and the risk of wheezing in the child at 1 and 3 years (Table 3). After multivariable adjustment, the previously observed significant inverse associations persisted (by age of 1: OR = 0.74 (95%CI = 0.47–0.92), *p* = 0.03; by age of 3: OR = 0.89 (95%CI = 0.61–1.00), *p* = 0.05). Furthermore, an analysis of maternal selenium concentration as a continuous variable revealed in the unadjusted models, a significant negative association between plasma selenium concentration and the risk of wheezing by 1 year (OR = 0.94 (95%CI = 0.90–0.99), *p* = 0.04) and a trend toward a negative association by 3 years (Table 3). However, the latter results were borderline‐significant (*p* = 0.06 by age of 1) after adjustment for potential confounding factors (Table 3).

**Table 3:** Association between maternal plasma selenium level and the child's risk of wheezing (*n* = 861)

	Maternal plasma selenium level (µg/L), OR [95%CI]*							
	Unadjusted				Adjusted^†^			
Outcome	High vs. low	*p* ^§^	For a 10 µg/L increment	*p*	High vs. low	*p*	For a 10 µg/L increment	*p*
Asthma								
By 1 year	0.93 [0.46–1.88]	0.46	0.99 [0.98–1.01]	0.50	0.99 [0.51–2.31]	0.89	1.00 [0.98–1.01]	0.77
By 3 years	0.73 [0.41–1.31]	0.31	0.97 [0.90–1.02]	0.07	0.83 [0.44–1.54]	0.67	0.99 [0.98–1.01]	0.30
Wheezing								
By 1 year	0.75 [0.49–0.94]	0.02	0.94 [0.90–0.99]	0.04	0.74 [0.47–0.92]	0.03	0.96 [0.92–1.01]	0.06
By 3 years	0.87 [0.61–0.99]	0.04	0.97 [0.90–1.02]	0.07	0.89 [0.61–1.00]	0.05	0.98 [0.93–1.04]	0.10
Atopic dermatitis								
By 1 year	1.33 [0.91–1.95]	0.18	1.00 [0.99–1.01]	0.25	1.44 [0.86–2.16]	0.62	1.00 [0.99–1.01]	0.24
By 3 years	0.98 [0.68–1.40]	0.04	1.00 [0.99–1.01]	0.78	1.17 [0.80–1.72]	0.30	1.00 [0.99–1.02]	0.73
Allergic rhinitis								
By 1 year	na		na		na		na	
By 3 years	0.96 [0.57–1.62]	0.04	1.00 [0.99–1.01]	0.74	0.99 [0.57–1.74]	0.63	1.00 [0.99–1.01]	0.51

*OR, odds ratio; CI, confidence interval.

^†^Adjusted for maternal age, pre‐pregnancy maternal BMI, tobacco use during pregnancy, exposure of the child to environmental tobacco smoke (from birth to 3 years of age), residence in damp housing (from birth to 3 years of age), maternal atopy, the child's gender, the season of birth, birthweight, exclusive breastfeeding for at least 4 months, the number of older siblings, the mother's educational level, and the household income; na: data not available.

^§^ *p*, *p*‐value.

There were no other significant relationships between selenium level (whether in tertiles or as a continuous variable) and the other health‐related variables by 1 and 3 years of age.

## Discussion

### Main findings

Our results showed a significant negative association between high maternal plasma selenium level during pregnancy and the child's risk of wheezing by the ages of 1 and 3. However, we did not find any associations between maternal plasma selenium level during pregnancy and asthma, allergic rhinitis, and atopic dermatitis. It may suggest that the disease prevalence is influenced by factors that could not be evaluated in this study, such as the child's diet and its exposure to other environmental factors.

Our results echo the few available literature reports of a beneficial effect of high fetal exposure to selenium on wheezing in infancy 8, 9, 10. Devereux et al. 8 evidenced a link between the pregnant woman's selenium status and the child's risk of wheezing at 2 years but not at 5 years. As in the present study, Thomson et al. 10 did not observe any association between maternal selenium status and the risks of asthma, allergic rhinitis and atopic dermatitis. Lastly, Shaheen et al. 9 did not find any association between fetal exposure to high selenium levels and postnatal atopic dermatitis.

### Potential mechanisms

There are three putative explanations for selenium's influence on the risk of wheezing during early childhood. Firstly, a number of animal studies have shown that maternal selenium deficiency is associated with impaired lung development 21. When the mother lacks selenium, oxidative stress is not adequately controlled and can therefore cause morphological and histological damage to the neonatal lung. Hence, selenium may have a beneficial effect on airway development and may protect against wheezing in infancy. In fact, it has been shown that an elevated risk of wheezing may be related to low airway caliber in the very early stages of postnatal life—a factor that can be decreased even further by viral infections or postnatal exposure to aeroallergens or tobacco smoke 22, 23. Secondly, it has been reported that selenium can modulate inflammatory and immune responses by influencing not only phagocytosis but also lymphocyte activation, proliferation and differentiation 24. A number of research studies have suggested that low selenium levels favor the differentiation of T‐helper (Th) cells towards the Th2 phenotype associated with asthma and allergic disease 25, 26, 27. Thirdly, the predominance of childhood wheezing associated with viral infections 28 prompted Broome et al. 25 to show that low selenium levels are linked to rapid viral replication and delayed viral clearance (due to a weak, delayed proliferative response by CD3+ T lymphocytes and low release of interferon gamma). Moreover, it has been demonstrated that changes in interferon gamma production during the first year of life predispose children to recurrent episodes of wheezing (from the preschool age up until adolescence) 29. Furthermore, selenium has a significant role in decreasing the oxidative stress induced by viral infection 30. Accordingly, it is possible that low maternal selenium levels affect the child's immune functions—making it vulnerable to viral infections and thus increasing the risk of respiratory symptoms in infancy.

Given the phenotypic and physiopathological heterogeneity of asthma, there is still no consensual definition of this condition in general or of childhood asthma in particular. Despite the fact that we did not find an association between maternal selenium status during pregnancy and asthma (as defined according to certain criteria) in the child under 3 years, we cannot conclude that selenium has no influence on the development of asthma during childhood and adolescence. Furthermore, the presence of wheezing in early infancy (often favored by viral infections and environmental factors such as tobacco smoke, aeroallergens, etc.) can contribute to the subsequent development of asthma in childhood and adolescence 31. In fact, many longitudinal studies have demonstrated that in most cases of asthma in adolescents and young adults, the first symptoms of the disease (such as wheezing) appeared during the preschool years 32, 33, 34. It has been also shown that 90% of cases of wheezing in infancy are associated with a viral infection of the respiratory tract 35.

Hence, by contributing to the alteration in the immune response that predisposes to bronchial obstruction during acute respiratory infections, selenium deficiency might be a risk factor for wheezing in the early postnatal period and for the subsequent development of asthma.

### Strengths and limitations

One of the strengths of our study was its measurement of selenium levels in the mother's blood during pregnancy, which reflects fetal exposure. In the general population, exposure to selenium is essentially dietary (i.e., via the ingestion of liquids and foods such as nuts, eggs, dairy products, poultry, meat, vegetables, etc.). Hence, maternal plasma selenium is a good marker of the mother's dietary intake of this oligo‐element. Most of the recent data on the role of selenium in human nutrition concern its beneficial effects on longevity 36, 37, cognitive function 38, and immune function 39. One study limitation relates to the fact that we did not assay selenium concentrations at several different time points during pregnancy. It would have been interesting to study the association between the health‐related variables and maternal selenium concentrations during the first trimester of pregnancy. In fact, the fetal lung begins to develop at the very start of the first trimester, with the embryonic and pseudoglandular phases 40. Secondly, we were unable to assay the plasma concentrations of glutathione peroxidases, selenoprotein P or heme oxygenase—all of which involved in the antioxidant defense system 41. Thirdly, we did not take into account the child's diet, with the exception of breastfeeding. Lastly, the child's health‐related data were reported by the parents on a questionnaire, which might have created classification bias. However, these questionnaires were based on validated ISAAC questionnaires, which doubtless helped to reduce this source of bias.

## Conclusion

The results of the present, prospective study suggest that the level of fetal exposure to maternal selenium could have an influence on the risk of wheezing in infancy and potentially on the risk of developing asthma later in life. This association, if causal, may have implications for primary prevention (e.g., dietary interventions during pregnancy). Until recently, predisposition to allergy was considered to be the main reason for the link between wheezing in early postnatal life and the subsequent development of asthma 42. There is now evidence to suggest that the susceptibility to viral infections (linked to selenium deficiency, amongst other things) is an additional risk factor for the link between wheezing in infancy and the development of asthma later in life 31. Lastly, a better understanding of the mechanisms and factors involved in the development of the asthma might provide us with new approaches for the prevention and treatment of asthma and other obstructive pulmonary diseases.

## Conflict of Interest

None declared.

## References

[1] Worldwide variations in the prevalence of asthma symptoms: the International Study of Asthma and Allergies in Childhood (ISAAC). 1998 Eur. Respir. J. 12(2):315–335. 9727780 10.1183/09031936.98.12020315

[2] Bousquet, J. , P. J. Bousquet , P. Godard , and J. P. Daures . 2005 The public health implications of asthma. Bull. World Health Organ. 83(7):548–554. 16175830 PMC2626301

[3] Gundacker, C. , S. Frohlich , K. Graf‐Rohrmeister , B. Eibenberger , V. Jessenig , D. Gicic , S. Prinz , K.J. Wittmann , H. Zeisler , B. Vallant , et al. 2010 Perinatal lead and mercury exposure in Austria. Sci. Total Environ. 408(23):5744–5749. 20825977 10.1016/j.scitotenv.2010.07.079

[4] Logdberg, B. , M. Berlin , and A. Schutz . 1987 Effects of lead exposure on pregnancy outcome and the fetal brain of squirrel monkeys. Scand. J. Work. Environ. Health 13(2):135–145. 3602968 10.5271/sjweh.2069

[5] Khanna, R. , and G. N. Johri . 1991 Lead and immunity: II. Suppression of humoral immune response to Hymenolepis nana in mice. J. Hyg. Epidemiol. Microbiol. Immunol. 35(1):1–7. 1715357

[6] Barceloux, D. G. 1999 Selenium. J. Toxicol. Clin. Toxicol. 37(2):145–172. 10382553 10.1081/clt-100102417

[7] Rudge, C. V. , H. B. Rollin , C. M. Nogueira , Y. Thomassen , M. C. Rudge , and J. O. Odland . 2009 The placenta as a barrier for toxic and essential elements in paired maternal and cord blood samples of South African delivering women. J. Environ. Monit. 11(7):1322–1330. 20449220 10.1039/b903805a

[8] Devereux, G. , G. McNeill , G. Newman , S. Turner , L. Craig , S. Martindale , F. Helms , A. Seaton . 2007 Early childhood wheezing symptoms in relation to plasma selenium in pregnant mothers and neonates. Clin. Exp. Allergy 37(7):1000–1008. 17581193 10.1111/j.1365-2222.2007.02757.x

[9] Shaheen, S. O. , R. B. Newson , A. J. Henderson , P. M. Emmett , A. Sherriff , and M. Cooke . 2004 Umbilical cord trace elements and minerals and risk of early childhood wheezing and eczema. Eur. Respir. J. 24(2):292–297. 15332400 10.1183/09031936.04.00117803

[10] Thomson, C. D. , K. Wickens , J. Miller , T. Ingham , P. Lampshire , M. J. Epton , G. I. Town , P. Pattemore , J. Crane ; Year six New Zealand Asthma and Allergy Cohort Study Group (NZAACS6). 2012 Selenium status and allergic disease in a cohort of New Zealand children. Clin. Exp. Allergy 42(4):560–567. 22417214 10.1111/j.1365-2222.2012.03924.x

[11] Heude, B. , A. Forhan , R. Slama , L. Douhaud , S. Bedel , M. J. Saurel‐Cubizolles , R. Hankard , O. Thiebaugeorges , M. De Agostini , I. Annesi‐Maesano , et al. 2016 Cohort Profile: the EDEN mother‐child cohort on the prenatal and early postnatal determinants of child health and development. Int. J. Epidemiol. 45(2):353–363. 26283636 10.1093/ije/dyv151

[12] Asher, M. I. , U. Keil , H. R. Anderson , R. Beasley , J. Crane , F. Martinez , E. A. Mitchell , N. Pearce , B. Sibbald , A. W. Stewart , et al. 1995 International study of asthma and allergies in childhood (ISAAC): rationale and methods. Eur. Respir. J. 8(3):483–491. 7789502 10.1183/09031936.95.08030483

[13] Ninan, T. K. , and G. Russell . 1992 Respiratory symptoms and atopy in Aberdeen schoolchildren: evidence from two surveys 25 years apart. BMJ. 304(6831):873–875. 1392746 10.1136/bmj.304.6831.873 PMC1882832

[14] Zannolli, R. , and G. Morgese . 1997 Does puberty interfere with asthma? Med. Hypotheses 48(1):27–32. 9049986 10.1016/s0306-9877(97)90020-7

[15] Patel, S. P. , A. Rodriguez , M. P. Little , P. Elliott , J. Pekkanen , A. L. Hartikainen , A. Pouta , J. Laitinen , T. Harju , D. Canoy , et al. 2012 Associations between pre‐pregnancy obesity and asthma symptoms in adolescents. J. Epidemiol. Community Health 66(9):809–814. 21844604 10.1136/jech.2011.133777 PMC3412048

[16] Sin, D. D. , S. Spier , L. W. Svenson , D. P. Schopflocher , A. Senthilselvan , R. L. Cowie , S. F. Man , et al. 2004 The relationship between birth weight and childhood asthma: a population‐based cohort study. Arch. Pediatr. Adolesc. Med. 158(1):60–64. 14706960 10.1001/archpedi.158.1.60

[17] Harley, K. G. , J. M. Macher , M. Lipsett , P. Duramad , N. T. Holland , S. S. Prager , J. Ferber , A. Bradman , B. Eskenazi , I. B. Tager , et al. 2009 Fungi and pollen exposure in the first months of life and risk of early childhood wheezing. Thorax. 64(4):353–358. 19240083 10.1136/thx.2007.090241 PMC3882001

[18] Knudsen, T. B. , S. F. Thomsen , C. S. Ulrik , M. Fenger , S. Nepper‐Christensen , and V. Backer . 2007 Season of birth and risk of atopic disease among children and adolescents. J. Asthma 44(4):257–260. 17530522 10.1080/02770900701246832

[19] Kurosaka, F. , T. Terada , A. Tanaka , Y. Nakatani , K. Yamada , J. Nishikawa , K. Oka , H. Takahashi , A. Mogami , T. Yamada , et al. 2011 Risk factors for wheezing, eczema and rhinoconjunctivitis in the previous 12 months among six‐year‐old children in Himeji City, Japan: food allergy, older siblings, day‐care attendance and parental allergy history. Allergol. Int. 60(3):317–330. 21502806 10.2332/allergolint.10-OA-0246

[20] Kramer, M. S. 2011 Breastfeeding and allergy: the evidence. Ann. Nutr. Metab. 59(Suppl 1):20–26. 22189253 10.1159/000334148

[21] Kim, H. Y. , M. F. Picciano , M. A. Wallig , and J. A. Milner . 1991 The role of selenium nutrition in the development of neonatal rat lung. Pediatr. Res. 29(5):440–445. 1896247 10.1203/00006450-199105010-00006

[22] Dezateux, C. , J. Stocks , I. Dundas , and M. E. Fletcher . 1999 Impaired airway function and wheezing in infancy: the influence of maternal smoking and a genetic predisposition to asthma. Am. J. Respir. Crit. Care Med. 159(2):403–410. 9927350 10.1164/ajrccm.159.2.9712029

[23] Martinez, F. D. 1997 Definition of pediatric asthma and associated risk factors. Pediatr. Pulmonol. Suppl. 15:9–12. 9316095

[24] Hoffmann, P. R. 2007 Mechanisms by which selenium influences immune responses. Arch. Immunol. Ther. Exp. (Warsz) 55(5):289–297. 18219759 10.1007/s00005-007-0036-4

[25] Broome, C. S. , F. McArdle , J. A. Kyle , F. Andrews , N. M. Lowe , C. A. Hart , J. R. Arthur , M. J. Jackson , et al. 2004 An increase in selenium intake improves immune function and poliovirus handling in adults with marginal selenium status. Am. J. Clin. Nutr. 80(1):154–162. 15213043 10.1093/ajcn/80.1.154

[26] Inglot, A. D. , J. Zielinska‐Jenczylik , E. Piasecki , L. Syper , and J. Mlochowski . 1990 Organoselenides as potential immunostimulants and inducers of interferon gamma and other cytokines in human peripheral blood leukocytes. Experientia. 46(3):308–311. 2107096 10.1007/BF01951774

[27] Jeong, D. W. , M. H. Yoo , T. S. Kim , J. H. Kim , and I. Y. Kim . 2002 Protection of mice from allergen‐induced asthma by selenite: prevention of eosinophil infiltration by inhibition of NF‐kappa B activation. J. Biol. Chem. 277(20):17871–17876. 11897787 10.1074/jbc.M200808200

[28] Martinez, F. D. , A. L. Wright , L. M. Taussig , C. J. Holberg , M. Halonen , and W. J. Morgan . 1995 Asthma and wheezing in the first six years of life. The Group Health Medical Associates. N. Engl. J. Med. 332(3):133–138. 7800004 10.1056/NEJM199501193320301

[29] Stern, D. A. , S. Guerra , M. Halonen , A. L. Wright , and F. D. Martinez . 2007 Low IFN‐gamma production in the first year of life as a predictor of wheeze during childhood. J. Allergy Clin. Immunol. 120(4):835–841. 17689598 10.1016/j.jaci.2007.05.050

[30] Schwarz, K. B. 1996 Oxidative stress during viral infection: a review. Free Radic. Biol. Med. 21(5):641–649. 8891667 10.1016/0891-5849(96)00131-1

[31] Martinez, F. D. 2009 The connection between early life wheezing and subsequent asthma: the viral march. Allergol. Immunopathol. (Madr.) 37(5):249–251. 19875225 10.1016/j.aller.2009.06.008

[32] Morgan, W. J. , D. A. Stern , D. L. Sherrill , S. Guerra , C. J. Holberg , T. W. Guilbert , L. M. Taussig , A. L. Wright , F. D. Martinez , et al. 2005 Outcome of asthma and wheezing in the first 6 years of life: follow‐up through adolescence. Am. J. Respir. Crit. Care Med. 172(10):1253–1258. 16109980 10.1164/rccm.200504-525OC PMC2718414

[33] Stern, D. A. , W. J. Morgan , M. Halonen , A. L. Wright , and F. D. Martinez . 2008 Wheezing and bronchial hyper‐responsiveness in early childhood as predictors of newly diagnosed asthma in early adulthood: a longitudinal birth‐cohort study. Lancet. 372(9643):1058–1064. 18805334 10.1016/S0140-6736(08)61447-6 PMC2831297

[34] Yunginger, J. W. , C. E. Reed , E. J. O'Connell , L. J. Melton, 3rd , W. M. O'Fallon , and M. D. Silverstein . 1992 A community‐based study of the epidemiology of asthma. Incidence rates, 1964–1983. Am. Rev. Respir. Dis. 146(4):888–894. 1416415 10.1164/ajrccm/146.4.888

[35] Jackson, D. J. , R. E. Gangnon , M. D. Evans , K. A. Roberg , E. L. Anderson , T. E. Pappas , M. C. Printz , W. M. Lee , P. A. Shult , E. Reisdorf , et al. 2008 Wheezing rhinovirus illnesses in early life predict asthma development in high‐risk children. Am. J. Respir. Crit. Care Med. 178(7):667–672. 18565953 10.1164/rccm.200802-309OC PMC2556448

[36] Akbaraly, N. T. , J. Arnaud , I. Hininger‐Favier , V. Gourlet , A. M. Roussel , and C. Berr . 2005 Selenium and mortality in the elderly: results from the EVA study. Clin. Chem. 51(11):2117–2123. 16123147 10.1373/clinchem.2005.055301

[37] Ray, A. L. , R. D. Semba , J. Walston , L. Ferrucci , A. R. Cappola , M. O. Ricks , Q. L. Xue , L. P. Fried , et al. 2006 Low serum selenium and total carotenoids predict mortality among older women living in the community: the women's health and aging studies. J. Nutr. 136(1):172–176. 16365078 10.1093/jn/136.1.172

[38] Akbaraly, T. N. , I. Hininger‐Favier , I. Carriere , J. Arnaud , V. Gourlet , A. M. Roussel , C. Berr , et al. 2007 Plasma selenium over time and cognitive decline in the elderly. Epidemiology. 18(1):52–58. 17130689 10.1097/01.ede.0000248202.83695.4e

[39] Rayman, M. P. 2002 The argument for increasing selenium intake. Proc. Nutr. Soc. 61(2):203–215. 12133202 10.1079/PNS2002153

[40] Kitterman, J. A. 1984 Fetal lung development. J. Dev. Physiol. 6(1):67–82. 6368667

[41] Lei, X. G. , W. H. Cheng , and J. P. McClung . 2007 Metabolic regulation and function of glutathione peroxidase‐1. Annu. Rev. Nutr. 27:41–61. 17465855 10.1146/annurev.nutr.27.061406.093716

[42] Martinez, F. D. 2003 Toward asthma prevention‐does all that really matters happen before we learn to read? N. Engl. J. Med. 349(15):1473–1475. 14534342 10.1056/NEJMe030041

