# Corrigendum

**DOI:** 10.1002/iid3.176

**Published:** 2017-08-24

Baïz, N., Chastang, J., Ibanez, G., & Annesi‐Maesano I. (2016). Prenatal exposure to selenium may protect against wheezing in children by the age of 3. *Immunity, Inflammation and Disease*, 5 (1), 37–44. DOI: 10.1002/iid3.138.

In the article “Prenatal exposure to selenium may protect against wheezing in children by the age of 3”, it has been noted that the ‘EDEN Mother‐Child Cohort Study Group was omitted in error. The correct author list for the article is shown below.

Nour Baïz^1^, Julie Chastang^1,2^, Gladys Ibanez^1,2^, Isabella Annesi‐Maesano^1^ and the EDEN Mother‐Child Cohort Study Group^3^.

^3^Members of the EDEN Mother‐Child Cohort Study Group includes the following members: I. Annesi‐Maesano, J. Y. Bernard, J. Botton, M.A. Charles, P. Dargent‐Molina, B. de Lauzon‐Guillain, P. Ducimetière, M. de Agostini, B. Foliguet, A. Forhan, X. Fritel, A. Germa, V. Goua, R. Hankard, B. Heude, M. Kaminski, B. Larroque, N. Lelong, J. Lepeule, G. Magnin, L. Marchand, C. Nabet, F. Pierre, R. Slama, M.J. Saurel‐Cubizolles, M. Schweitzer, O. Thiebaugeorges.

We apologize for any inconvenience caused.

